# Preparation of Nano-Apatite Grafted Glass-Fiber-Reinforced Composites for Orthodontic Application: Mechanical and In Vitro Biofilm Analysis

**DOI:** 10.3390/ma15103504

**Published:** 2022-05-13

**Authors:** Abdul Samad Khan, Alaa Alshaia, AlAnood AlDubayan, Sundus Alarifi, Abdulaziz Alamri, Hanan Aldossary, Syed Zubairuddin Ahmed, Ijlal Shahrukh Ateeq, Abbas Saeed Hakeem, Suriya Rehman

**Affiliations:** 1 Department of Restorative Dental Sciences, College of Dentistry, Imam Abdulrahman Bin Faisal University, Dammam 31441, Saudi Arabia; szahmed@iau.edu.sa; 2 College of Dentistry, Imam Abdulrahman Bin Faisal University, Dammam 31441, Saudi Arabia; alaalshaia@gmail.com (A.A.); andubayan@gmail.com (A.A.); sundusalarifi@hotmail.com (S.A.); 3 Department of Preventive Dental Sciences, College of Dentistry, Imam Abdulrahman Bin Faisal University, Dammam 31441, Saudi Arabia; absalamri@iau.edu.sa; 4 Department of Epidemic Diseases Research, Imam Abdulrahman Bin Faisal University, Dammam 31441, Saudi Arabia; haalhameem@iau.edu.sa; 5 Biomedical Engineering Department, College of Engineering, Imam Abdulrahman Bin Faisal University, Dammam 31441, Saudi Arabia; lsateeq@iau.edu.sa; 6 Interdisciplinary Research Center for Hydrogen & Energy Storage (IRC-HES), King Fahd University of Petroleum and Minerals, Dhahran 31261, Saudi Arabia; ashakeem@kfupm.edu.sa

**Keywords:** orthodontics, glass fibers, hydroxyapatite, nano-particles, grafting, retainers, bond strength, fatigue testing, antibacterial, biofilm

## Abstract

This study aimed to fabricate nano-hydroxyapatite (nHA) grafted/non-grafted E-glass-fiber-based (nHA/EG) and E-glass fiber (EG) orthodontic retainers and to compare their properties with commercially available retainers. Stainless-steel (SS) retainers and everStick Ortho (EST) were used as control groups. The retainers were evaluated with Raman spectroscopy and bonded to bovine teeth. The samples were fatigued under cyclic loading (120,000 cycles) followed by static load testing. The failure behavior was evaluated under an optical microscope and scanning electron microscope. The strain growth on the orthodontic retainers was assessed (48h and 168h) by an adhesion test using *Staphylococcus aureus* and *Candida albicans*. The characteristic peaks of resin and glass fibers were observed, and the debonding force results showed a significant difference among all of the groups. SS retainers showed the highest bonding force, whereas nHA/EG retainers showed a non-significant difference from EG and EST retainers. SS retainers’ failure mode occurred mainly at the retainer–composite interface, while breakage occurred in glass-fiber-based retainers. The strains’ adhesion to EST and EG was reduced with time. However, it was increased with nHA/EG. Fabrication of nHA/EG retainers was successfully achieved and showed better debonding force compared to other glass-fiber-based groups, whereas non-linear behavior was observed for the strains’ adhesion.

## 1. Introduction

Orthodontic treatment provides facial esthetics and improves dental health, which can be achieved by proper teeth alignment. Retention is needed after orthodontic treatment to avoid relapse after the final occlusal outcome [1]. Relapse can happen due to the forces of periodontal fibers and can occur because of deflecting occlusal contacts if the occlusion was not ideally positioned. The stability of the orthodontic treatment can be affected by age through dentofacial growth, and changes occur in the surrounding soft tissue [2]. The elastic fibers around the neck of teeth and dento-gingival and interdental fibers take the longest to remodel, which requires about eight months or more [3]. Orthodontic retainers have been used to hold teeth for the period necessary to maintain the result [4]. Fixed retainers offer the advantage of being placed permanently and eliminate the need for patient compliance. They are typically bonded to the lingual/palatal surface of the labial segment. Fixed retainers are more prone to plaque and calculus accumulation, as they cannot be removed for cleaning [5]. It is also mandatory to ensure that fixed retainers are still bonded in their appropriate place. The main factors defining the success and longevity of lingual retainers are the type of composite resin used for bonding, the type of retainer material, the location of the retainer (i.e., maxillary or mandibular arch), and the number of units included for bonding [6].

The most commonly used materials for dental retainers are stainless-steel wires with variable integral properties and stiffness [7]. Previous studies have indicated acceptable compatibility of metal-bonded retainers with periodontal health [8,9] and showed success rates between 60–90% in long-term usage [10]. However, the multi-stranded bonded lingual retainer has some esthetic limitations and usage restrictions in nickel allergy patients [11]. Therefore, fiber-reinforced composite (FRC) retainers are used to substitute stainless-steel wires, and hence, they are metal-free alternatives, have higher esthetic, and allow chemical adhesion of the retainer to the bonding agent [12]. The most common causes of lingual retainer failures are failure of the retainer–composite interface, detachment of resin pads at the composite–enamel interface, and breakage of the retainer [13,14]. Deficient bonding procedures, such as inadequate moisture control or mishandling of the resin material, are reasons for debonding of the resin pad from the tooth [15]. Studies have reported variations of 49–95% in the survival rate of FRC as an orthodontic retainer [16,17]. The disagreement among such studies implies that further research for retainer comparisons is necessary.

The initial adhesion of bacteria to an orthodontic retainer is the critical factor in forming pathological biofilms [18,19]. These biofilms are hard to entirely remove with a toothbrush and are the leading cause of dental caries and periodontal diseases [20]. Thus, it is essential to prevent the adhesion of pathological biofilms before it occurs. The presence of bacteria on an orthodontic retainer can increase carious lesion and periodontal disease risks.

Particulate hydroxyapatite (HA) is known for its biocompatibility and is widely used as biomimetic material due to its structural similarity to enamel [21]. Various studies have proved the efficacy of HA particles in caries prevention and in improving periodontal disease [22,23]. Recently, short-cut nano-HA grafted E-glass fibers have been incorporated into dental resins and showed improved mechanical properties, in vitro bioactivity, bio-adhesion to the tooth structure, and cell compatibility [24,25,26]. Our previous study [27] evaluated the flexural strength/modulus, 3-D structure, and morphological analysis of nano-HA/glass-fiber-reinforced resins. However, the authors could not find any study where nano-hydroxyapatite (nHA)-incorporated glass-fiber-based orthodontic retainers have been used as retainers. There is a lack of evidence that describes the mechanical properties under fatigue stress and the antibacterial activities of orthodontic retainer surfaces coated with nHA. Therefore, the aim of this study was to fabricate nHA/glass-fiber-reinforced resin-based retainers, perform structural analysis, analyze the fatigue resistance and debonding force, and also investigate the bacterial and fungal adhesion behavior. The hypothesis of this study was that the debonding force of the nHA grafted glass-fiber-based retainer would be higher than that of the non-grafted glass fibers and commercially available glass-fiber-based retainers. Furthermore, it was hypothesized that the nHA grafted glass-fiber-based retainers would show reduced bacterial and fungal growth. Thus, the null hypothesis was that the debonding force of the nHA grafted glass-fiber-based retainers would be less than that of the non-grafted glass fibers and commercially available glass-fiber-based retainers. Furthermore, the null hypothesis stated that nHA grafted glass-fiber-based retainers would show increased bacterial and fungal growth on the surface.

## 2. Materials and Methods

### 2.1. Synthesis of Grafted and Non-Grafted Fibers

The chemicals used in this study were purchased from Sigma Aldrich, St. Louis, MO, USA, and were of analytical grade. The E-glass fibers were activated, and the microwave irradiation technique was used to synthesize nano-hydroxyapatite (nHA) grafted fibers, as described previously [27]. However, in this study, the E-glass fibers were cut to 25 mm length.

### 2.2. Silanization of Fibers

The silanization of obtained nHA/E-glass fibers and non-coated E-glass fibers (EG) was carried out using 3-(trimethoxysilyl)propyl methacrylate (MPS (SHBJ1178; Sigma Aldrich, Shanghai, China)), where 1.0 vol. % solution of MPS was prepared by using a solvent mixture of 90% ethanol and 10% deionized water. The pH of the solution was adjusted to 4 with 3.0 M acetic acid (J2550; Honeywell GmbH, Regen, Germany). The silane solution was stirred (Benchmark, Sayreville, NJ, USA) and allowed to hydrolyze for 1 h. Then, the mixture was filtered, washed with absolute ethanol (K50375083828; EMSURE^®^, Darmstadt, Germany), and left overnight at room temperature to dry. Later, the filtrate was dried at 60 °C for 72 h. Then, the samples of both batches were stored in a desiccator to avoid moisture contamination. The structural and morphological patterns of silanized fibers were analyzed by Fourier transform infrared spectroscopy (FTIR; Thermo Fisher Scientific, Waltham, MA, USA) and scanning electron microscopy (SEM; Tescan Vega 3, Brno-Kohoutovice, Czech Republic), as described (images provided in Supplementary Materials, Figure S1) in detail previously [28].

### 2.3. Fabrication of Experimental Fiber Posts

The resin matrices were mixed at ambient room temperature, and the ratio of resin matrices, i.e., bisphenol dimethacrylate (*bis*-GMA; MKCD8912; Sigma-Aldrich, St. Louis, MO, USA) and urethane dimethacrylate (UDMA; MKCD6166; Sigma-Aldrich, St. Louis, MO, USA), was set at 70:30, respectively. These monomers at calculated weight percentages as per their ratio were mixed for 30 min at 45 °C. Then, 0.5 wt. % camphorquinone (09003AQV; Sigma-Aldrich) and 0.5 wt. % co-initiator ethyl 4-(dimethylamino) benzoate (MKBX1335V; Sigma-Aldrich, St. Louis, MO, USA) were added, and the reaction was allowed to stir for 30 min at 25 °C. The reaction was conducted in a dark environment to avoid premature polymerization. The silanized non-coated E-glass fibers and nHA/E-glass fibers were then separately placed unidirectionally in an experimental resin matrix, where the fiber–resin matrix ratio was set at 50 wt. %. Fibers from both groups were tightly packed and condensed with a flat-end condenser and allowed to immerse completely in the resin matrix. The prepared un-cured fiber-based orthodontic retainers were foiled in aluminum folds to avoid premature polymerization. Twisted bondable lingual retainer (3M, St. Paul, MN, USA) and EverStick Ortho (Sticktech Ltd., Turku, Finland) were used as the control groups. Table 1 shows the description of the control and experimental materials.

After receiving approval from the research unit committee, College of Dentistry, Imam Abdulrahman Bin Faisal University, Dammam, Saudi Arabia, a total of 80 extracted caries-free bovine mandibular incisor teeth with intact lingual enamel were collected. The sample size was calculated as per World Health Organization’s specifications to obtain a study power equal to 90% and a level of significance equal to 5% [29], and the minimum sample size was calculated (n = 10) according to a previous study [30]. Teeth were initially sterilized with 70% ethanol solution for 10 min, then cleaned from debris, and stored in 1% thymol solution at 4 °C until prepared.

### 2.4. Preparation and Grouping of the Samples

**Figure 1:**
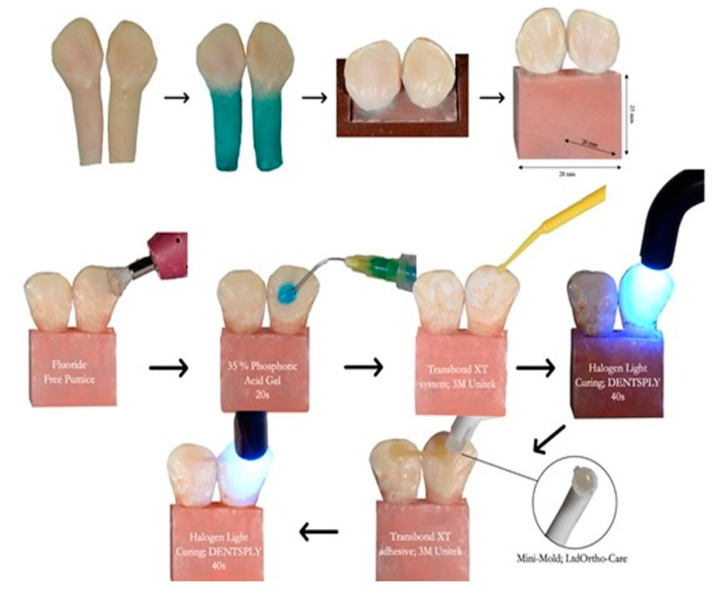
Molding of teeth, preparation, and placement of retainer on bovine teeth.

For mechanical testing, the study design was adopted from Cooke and Sherriff [31]. Silicon mold with dimensions of 28 mm width × 20 mm depth × 25 mm height was used to construct self-cured acrylic resin blocks. To mimic the periodontal structure, a thin layer of self-curing silicone (3M, Maplewood, MN, USA) was painted over the root up to the cemento-enamel junction [32]. A total of 40 samples were prepared by mounting a pair of mandibular incisors in self-curing acrylic until the cervical margin matched in contact points and its long axis was perpendicular to the base of molds, as shown in Figure 1. Then, 10 specimens were randomly assigned to each group. For the preparation for bonding, a fluoride-free pumice was used to clean the enamel surface, followed by acid etching with 35% phosphoric acid gel (FineEtch, Spident Co., Ltd., Little Ferry, NJ, USA) for 30 s, followed by washing and drying. Then, primer (Transbond XT system; 3M Unitek, Monrovia, CA, USA) was applied. A retainer with a length of 15 mm of each material was located between two adjacent teeth and bonded with light cure adhesive (Transbond XT adhesive; 3M Unitek). A dome-shaped wire bonder tip (Mini-Mold™; Ortho-Care Ltd., Bradford, West Yorkshire, UK) was used to standardize the amount of composite used for bonding, followed by light curing for 60 s (Halogen Curing Light, Dentsply Sirona, Charlotte, NC, USA). After 60 min, the specimens were placed in deionized water at 37 °C for 22 h and 2 h in dry mode at 37 °C before testing.

### 2.5. Characterizations

#### 2.5.1. Raman Spectroscopy

The control and experimental samples’ structural patterns were analyzed using a DXR2 Raman microscope (Thermo Fischer Scientific, Waltham, MA, USA), whereby point and line Raman spectra were taken. A spectral resolution of 2 cm^−1^ with an excitation wavelength of 532 nm laser was used, and the laser power used was 30 mW. The spectra were acquired at 25 °C between 200 and 3500 cm^−1^.

#### 2.5.2. Cyclic Loading and Debonding Force Testing

An intermittent vertical force was delivered to the specimen 120,000 times with a frequency of 2 Hz. The vertical dynamic force was applied using 1.0 mm × 1.0 mm jig attached to a fatigue-testing machine (ElectroPuls™ E3000, Instron, Norwood, MA, USA). The midpoint of the interdental retainer segment was fatigued, and after completing the cycles, the surface morphology was recorded with an optical microscope (ELMSFORD 10523, LUXO, New York, NY, USA) at 10×, 25×, and 40×. Then, selected samples were evaluated by using a scanning electron microscope (SEM) (VEGA-3 LMU; Tescan, Czech Republic) at 15 kV.

After fatigue testing, the vertical load was applied at the midpoint of the interdental retainers at a crosshead speed of 1 mm·min^−1^ to simulate the bite force. The load applied to the wire was gradually increased until debonding occurred, and the force was recorded in Newtons (N).

#### 2.5.3. Fracture Mode Analysis

The failure of the bond or fiber was initially evaluated under an optical microscope, and then representative samples were taken for SEM analysis. For SEM analysis, the samples were gold coated (Quoram Technologies, Lewes, UK) for 90 s, and morphological images were taken at different magnifications. Furthermore, the failure was classified as follows [15]:

- Type 1: complete adhesive debonding of the retainer from the tooth surface.
- Type 2: partial adhesive detachment of the retainer from one of the teeth.
- Type 3: retainer did not debond from the tooth surface but fractured.
- Type 4: retainer did not debond from the tooth surface, but the overlying composite detached.
- Type 5: Combination of more than one type.

#### 2.5.4. Bacterial and Fungal Growth Analysis

##### Preparation of Inoculum

The bacterial strain *Staphylococcus aureus* ATCC^®^ 25923™ and fungal strain *Candida albicans* ATCC^®^ 14053™ (yeast) were selected for biofilm formation on the prepared control and experimental retainers. *S. aureus* and *C. albicans* were aerobically grown in brain heart infusion (BHI) broth and RPMI 1640 culture medium, respectively, at 37 ± 2 °C for 24 h with shaking at 150 rpm. Subsequently, the cells were harvested and washed using phosphate buffer saline (PBS), and cell density was adjusted to approximately 10^7^ CFU/mL.

##### Biofilm Assay

Biofilm on the processed orthodontic retainers was evaluated by the adhesion test. Bacterial and fungal biofilm formation on processed samples and control groups was carried out in sterile 12-well plates. Each sample group was placed in the well, contacting 1 mL of the *C. albicans* and *S. aureus* suspension with CFU of 10^7^ per milliliter of broth so that the samples were submerged completely. Plates were incubated at 37 ± 2 °C with shaking at 150 rpm for two sets of incubation periods, i.e., 48 h and 168 h (7 days). Two processed pieces and two control pieces were used for each strain. The freshly prepared growth medium was regularly replaced for the 7-day experimental set.

The capability of colony formation by adhered cells was assessed by plate count method and was carried out as follows: The sample piece was taken out from the suspension and rinsed thrice using PBS to get rid of non-adherent cells. Subsequently, the samples were transferred to a new plate containing 1 mL of sterile 0.1% TritonX-100 PBS solution. The plates were subjected to sonication for 10 min to obtain the sample surface’s adherent cells. An aliquot of 100 µL of the cell suspension was taken and diluted using 10-fold serial dilution with sterile normal saline, whereby 100 µL was plated out by spread plate technique using Sabauraud Dextrose Agar (SDA) and BHI agar for *Candida* and *S. aureus,* respectively.

The plates were incubated at 37 ± 2 °C, and the number of adherent cells via colony formation determined the biofilm formation after 48 h and 168 h for both strains. Results were recorded by manual colony counting, and photographs were taken. Each colony counted on the plate was considered to have emerged from a single viable microbial cell. The biofilm rate (B_R_) was expressed by adherent cell percentage, evaluated by using the following formula: B_R_ % = (A/B) × 100% (where A is the number of CFUs in the medium treated with nanomaterial, and B is the number of CFUs in control).

### 2.6. Statistical Analysis

Statistical analysis was carried out using SPSS software (IBM Software, Armonk, NY, USA) version 22. The Shapiro–Wilk test was used to test the normal distribution of data. Means were analyzed by one-way analysis of variance (ANOVA) post hoc Tukey’s test, and p-values less than 0.05 were considered significant.

## 3. Results

### 3.1. Raman Spectroscopy

**Figure 2:**
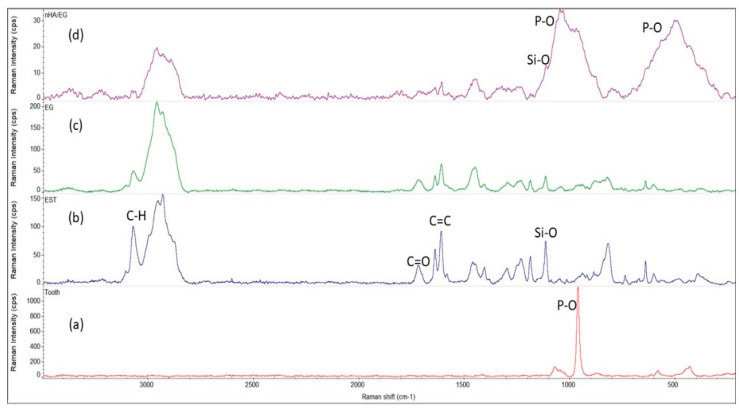
Comparative Raman spectra of (**a**) tooth, (**b**) EST, (**c**) EG, and (**d**) nHA/EG. The characteristic stretching P-O peak appeared at 963 cm^−1^. The glass-fiber-based retainers showed stretching C-H peaks (3100–2800 cm^−1^), C=O (1724 cm^−1^), aliphatic (164 cm^−1^), aromatic (1610 cm^−1^), and Si-O (1120 cm^−1^). The nHA/EG showed the presence of phosphate peaks.

Comparative Raman spectra of the tooth, EST, EG, and nHA/EG are shown in Figure 2a–d, wherein Figure 2a shows the characteristic peaks of the tooth structure. Phosphate peaks *v_3_* were observed at 1080 cm^−1^ and 1050 cm^−1^, and the *v_1_* stretching peak was observed at 963 cm^−1^, which was dominant in the sample spectrum. The *v_4_* and *v_2_* phosphate bending peaks were observed at 584 cm^−1^ and 434 cm^−1^. Figure 2a–c shows an asymmetric stretching peak of –CH_3_ at 3070 cm^−1^ and 2961 cm^−1,^ and symmetric stretching peaks of –CH_3_ appeared at 2934 cm^−1^ and 2890 cm^−1^. The carbonyl (C=O) appeared at 1724 cm^−1^, and peaks at 1640 cm^−1^ and 1610 cm^−1^ were attributed to aliphatic and aromatic stretching, respectively, from *bis*-GMA. A weak CH_2_ peak was observed at 1465 cm^−1^, and the 1408 cm^−1^ peak was attributed to the benzene ring’s strong v(C–C). The Si-O peak appeared at 1120 cm^−1^. Figure 2d shows a broad band of the P-O group at 1060–960 cm^−1^.

### 3.2. Mechanical Testing

**Figure 3:**
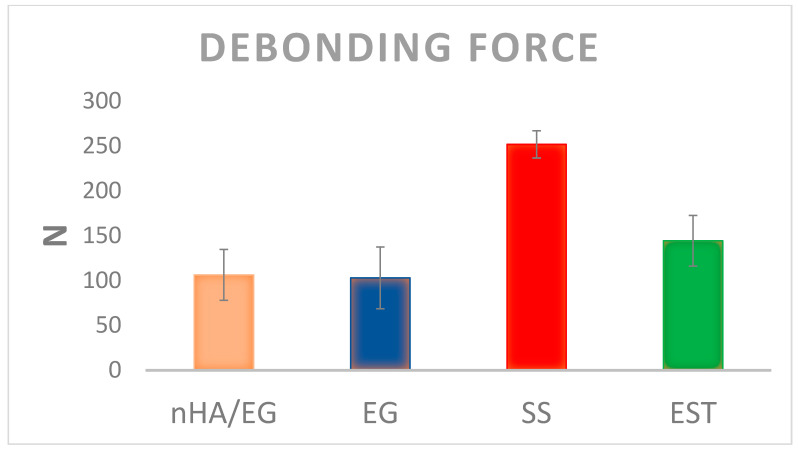
Mean and standard deviation of the debonding force of bonded control and experimental lingual retainers.

**Figure 4:**
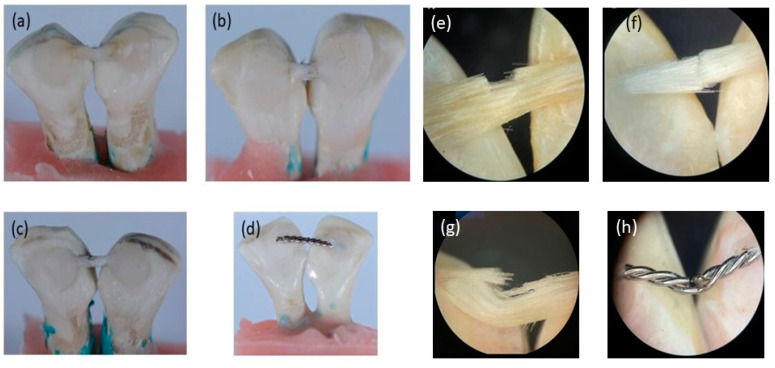
Microscopic images at different magnifications of (**a**,**e**) nHA/EG, (**b**,**f**) EG, (**c**,**g**) EST, and (**d**,**h**) SS retainers showing the retainer’s surface after cycling load (120,000 cycles) in compression mode.

**Figure 5:**
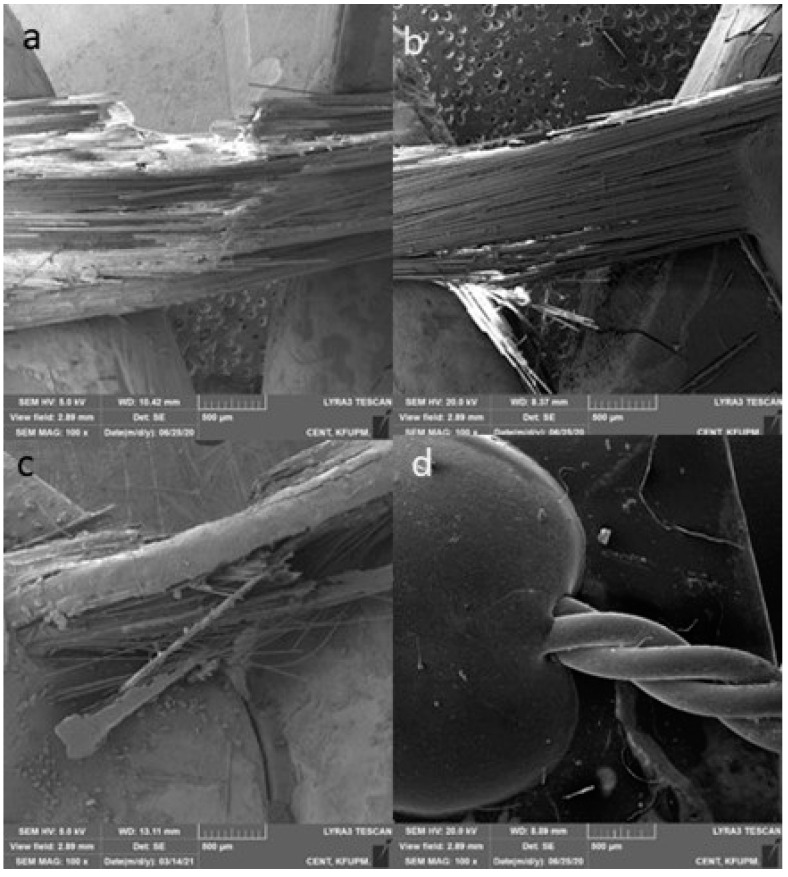
Representative images of (**a**) nHA/EG, (**b**) EG, (**c**) EST, and (**d**) SS retainers after fatigue testing.

**Figure 6:**
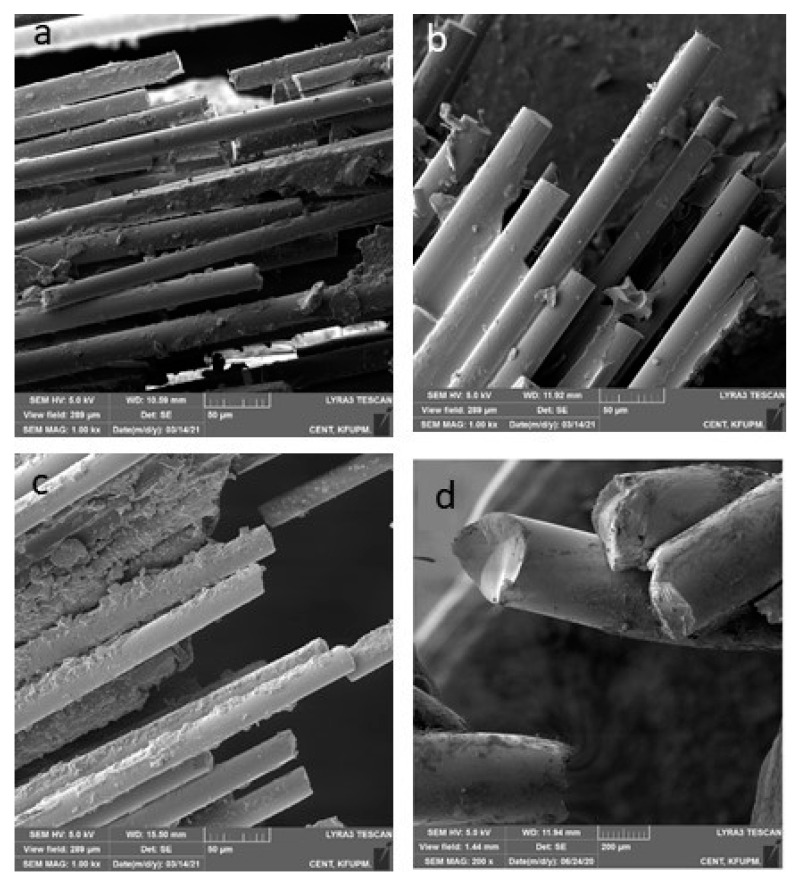
Representative scanning electron microscopic images of (**a**) nHA/EG, (**b**) EG, (**c**) EST, and (**d**) SS retainers after static loading showing the fractured parts of the retainers.

The mean debonding forces of nHA/EG, EG, EST, and SS retainers are given in Figure 3. The SS retainers showed a significantly higher debonding force when compared to nHA/EG retainers (*p*-value = 0.01), EG retainers (*p*-value = 0.003), and EST retainers (*p*-value = 0.005). The debonding force did not differ significantly between fiber-based retainer groups (nHA/EG vs. EG vs. EST). The difference of nHA/EG was non-significant compared to the EG retainers (*p*-value = 0.27) and EST retainers (*p*-value = 0.72). The optical microscopic and SEM images after cyclic loading showed that none of the specimens fractured during cyclic loading, as shown in Figure 4 and Figure 5. However, surface damage was revealed, and the EST retainer’s morphological pattern revealed more damage than the other groups. Based on optical microscopic (Figure 4) and the SEM images (Figure 6), the failure types for the fiber-reinforced retainers and stainless-steel retainers after debonding are presented in Table 2. The observed failure behaviors for the fiber-reinforced-based retainer groups were type 3 and a combination of types 3 and 4. The failure types in the stainless-steel group were categorized equally into types 1 and 2. The SEM images revealed the fractured patterns of retainers after debonding, and it was observed that the glass fibers were impregnated in the resin matrix.

### 3.3. Bacterial and Fungal Growth Analysis

**Figure 7:**
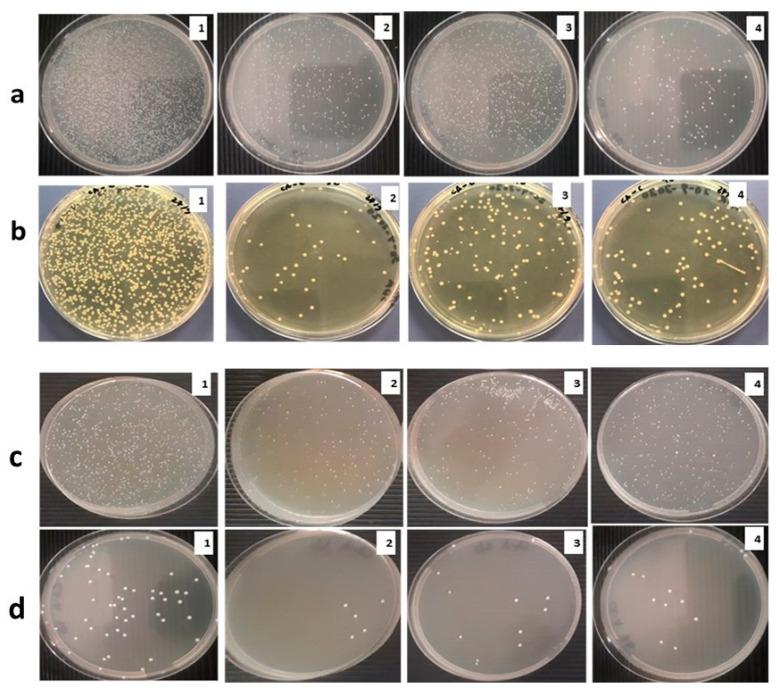
Macroscopic observations of adherent cell count of (**a**) *S. aureus* and (**b**) *C. albicans* after incubation for 48 h and (**c**) *S. aureus* and (**d**) *C. albicans* after incubation for 168 h. (1: SS; 2: EST; 3: nHA/EG; 4: EG).

Biofilms on the processed orthodontic retainers were analyzed by the cell adhesion test. The results obtained are presented in Figure 7, which clearly shows that the number of colonies on the plates inoculated with processed orthodontic retainers decreased markedly compared to SS.

**Figure 8:**
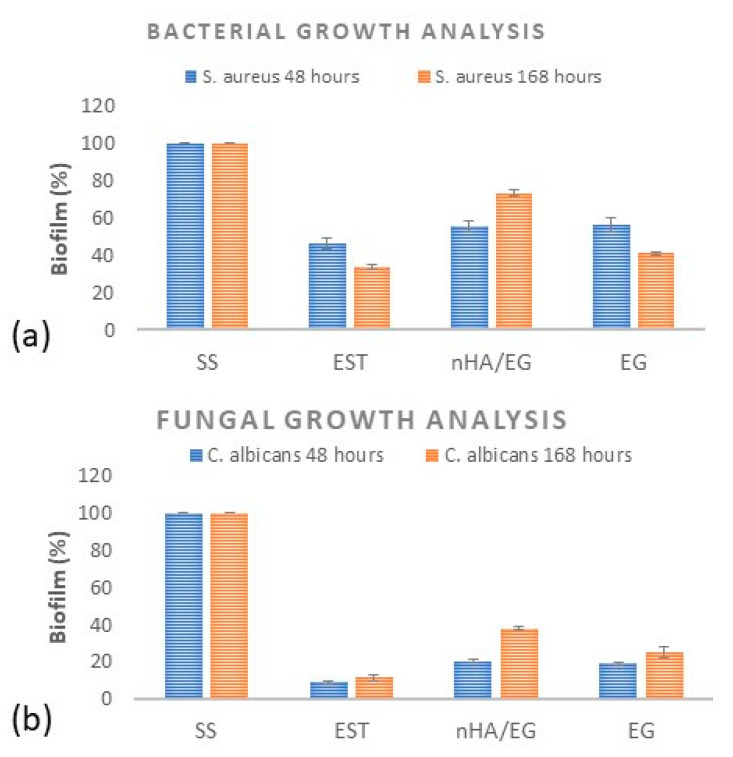
Rate of biofilm formation ((**a**) *S*. *aureus* (**b**) *C*. *albicans*) on processed orthodontic retainers and controls measured by counting colony-forming units (CFU) after incubation for 48 h and 168 h.

Additionally, it was found that after incubation for 48 h, the B_R_ of the SS retainers and EST was 43% and 8.7%. Whereas for the nHA/EG retainers, B_R_ was 56% and 21%, for the EG retainers, B_R_ was 58% and 19.2% against *S. aureus* and *Candida,* respectively (Figure 8a,b). For *S. aureus*, a statistical difference was found between EST and nHA/EG (*p* = 0.023) and EST and EG (*p* = 0.027). However, a non-significant difference was found between nHA/EG and EG (*p* = 0.74). For *C. albicans*, a statistically significant difference (*p* < 0.05) was found. Another set of experiments in which the incubation period was 7 days showed a similar pattern of the B_R_ of the SS retainers and EST retainers, which were 32.5% and 11%. Whereas for the nHA/EG retainers, B_R_ was 73% and 37.7%, for the EG retainers, B_R_ was 42% and 26.75% against *S. aureus* and *Candida*, respectively. A statistically significant difference was observed between *S. aureus* and *C. albicans*. All groups showed a significant difference between 48 h and 168 h.

## 4. Discussion

Based on the results of this study, the null hypothesis was partially rejected, as a non-significant difference was observed for the debonding force among the glass-fiber-based retainers. The nHA grafted glass-fiber-based retainers showed a non-significant difference in bacterial and fungal strain growth compared to non-grafted glass-fiber-based retainers. However, significantly higher values were observed compared to commercially available glass-fiber-based retainers. The data are summarized in Table S1.

Orthodontic retainers are commonly used to ensure steady and stable retention after orthodontic treatment. The main advantage of fixed retention is eliminating the need for patient compliance, which is crucial in the case of using removable retainers. However, fixed retention has several shortcomings, including the risk of breakage and failure of the retainers [33]. In addition, the orthodontic retainer acts as a gathering site for bacteria, biofilm, and dental calculus, which cause caries and periodontal disease [34,35]. Thus, it is vital to enhance the bonding force of the orthodontic retainer and prevent pathogenic bacteria from adhering to it.

Many studies have investigated the effect of adding nHA on the mechanical properties of dental polymers and found improved values [36,37,38]. Similarly, the results of this study revealed that the nHA grafted fiber-based retainer showed a non-significant difference in debonding force compared to pure E-glass fiber and everStick retainers. This might suggest that adding silanized nHA to fiber-reinforced composite orthodontic retainers might enhance the fatigue resistance. It is anticipated that the presence of OH on the surface of nHA is linked with the carbonyl group of silane coupling agents. The surface charges and surface potential of nHA can influence the interaction [39]. The interaction of the organic structure with apatite crystals is due to the electrostatic binding of the carbonyl group and surface mineral phosphate groups via calcium ions, which have been discussed previously [40]. In the present study, a combination of *bis*-GMA and UDMA was used and appropriately mixed to chemically and physically crosslink the two resins to form a semi-interpenetrating polymeric network. It has been reported that UDMA has the ability to form a hydrogen bond with *bis*-GMA, and it is strong enough to increase the mechanical properties of dental composites [41]. UDMA resins have high reactivity, high glass transition, greater flexibility, and higher morphological homogeneity [42]. Triethylene glycol dimethacrylate (TEGDMA) was not used in the composition, as it involves only physical crosslinking and does not contribute to network formation, leading to inhomogeneous polymer formation. It has high hydrophilic properties and is susceptible to cyclization and polymerization shrinkage [43,44].

Bonded lingual retainers are clinically exposed to various cyclic stresses from mastication, occlusion, and intraoral habits. The repetition of light forces results in fatigue formation and might lead to the partial or complete failure of retainers. Although in most cases, these forces do not reach the maximum debonding forces in an in vitro setting, they may fail due to the destructive effect of high-magnitude forces that rarely happen clinically. Hence, fatigue tests are better able to predict clinical durability than static tests [45]. The Newton unit was chosen to represent the fatigue testing results, as it reflects the unit of force, as opposed to Pascal, which represents the unit of pressure. If the Pascal unit were used, it would suggest that the force was equally distributed over the area of bonding, which was rejected in a bracket loading study [46]. In fact, different types of forces might be generated at both ends of the retainer complex when subjected to a vertical force, and tension, shear, and torsion forces might happen at once [38]. In the present study, the clinical bite was simulated by applying a vertical force, as it has been found that a vertical force yields the highest values of shear force compared to a tensile force in horizontal or vertical orientation [47]. Furthermore, the point of application has an effect on the shear force. It has been shown in the literature that the lowest values of shear bond strength are obtained when the force is applied to the interdental segment [48]. Therefore, this weak portion was chosen to determine the minimum force required for debonding.

The mean debonding force results did not significantly differ between the reinforced composite groups, although their compositions differed. Since the adhesion between the fiber and composite is chemical while that of the stainless-steel wire is mechanical, it was anticipated that the fiber-reinforced composite groups would show higher debonding force than the stainless-steel wire. However, despite their superior esthetics, the fiber-reinforced composite retainers showed statistically significantly lower values than stainless steel retainers did. This might be attributed to the anisotropic behavior of the fiber-reinforced composite and its relatively poor ability to absorb energy resulting from local impact damage [49]. This finding is consistent with Foek et al.’s study [13], which aimed to compare the shear bond strength of stainless-steel retainers versus fiber glass retainers and found that stainless steel retainers showed the highest bond strength. However, fiber glass retainers proved to be easy to handle and do not require adjustment to fit bonded teeth, contrary to stainless-steel retainers.

An orthodontic material must resist forces of 6–8 N to be suitable for a clinical application [50]. In the present study, all of the tested retainers showed higher values of debonding force; therefore, it is expected to show a decent clinical performance. Commonly, clinicians aim for their retainers to last between 6–12 months. Therefore, in this study, the load cycling was about 120,000 cycles, equivalent to 6 months of clinical service [51].

Nowadays, nHA is used broadly in dental fields to remineralize the diseased tooth structure and inhibit bacterial growth [52]. The antibacterial properties of nHA are related and proportional to its ability to release ions such as calcium and phosphate, which play a role in controlling dental plaque formation [53]. In the current study, the antibiofilm properties of processed orthodontic retainers were examined against Gram-positive *S. aureus and the yeast C. albicans* by reviewing the number of adherent cells. The cell suspensions (1 × 10^7^ CFU/mL) were kept in direct contact with processed orthodontic retainers and control samples for 48 h and 168 h at 37 °C. After incubation, 100 µL of each strain suspension was plated evenly on its specific agar plate for specific incubation parameters, and the numbers of colonies were counted. Thereafter, it was observed that the numbers of adherent cells of *S. aureus* and *C. albicans* colonies on the stainless-steel retainers were higher than those on the EST and the nHA/EG and EG retainers. This study used *S. aureus* and *C. albicans* to evaluate the growth of the strains on orthodontic retainers. Though *Streptococcus mutans* is known to cause primary caries [54], various studies on the oral cavity among healthy children and adults reported that the prevalence of *S. aureus* was in the range of 33% to 64% and 4% to 36%, respectively [55,56]. Another study showed that the prevalence of *S. aureus* in the oral cavity was in the range of approximately 33% in dental plaque and 47% in saliva [57]. Similarly, *C. albicans* is the most common type of fungus in the oral cavity [58]. Both *S. aureus* and *C. albicans* tend to adhere to the dental prosthesis and are opportunistic species that cause dental infections [58,59]. Therefore, these two strains were selected in this study.

The clinical significance of this study is that nHA has become one of the most widely studied materials that promote hard tissue regeneration in many dental specialties and is of particular importance in orthodontics because of its biocompatibility and regenerative and antimicrobial properties. The limitations of the present study are related to the bovine teeth used. However, Feagein et al. [60] reported that the Ca/P ratio of bovine teeth is similar to that of human tooth enamel after remineralization and demineralization. The bovine teeth selected had different dimensions, which could affect the length of the interdental segment. Additionally, the short length of the retainer compared to the clinical situation could affect the results.

## 5. Conclusions

Within the limitation of this laboratory-based study, it is concluded that the fabrication of experimental retainers was successfully conducted using silanized E-glass fibers (EG) and nano-hydroxyapatite grafted E-glass fibers (nHA/EG). Both types of fibers were impregnated in resin matrices. The debonding force of both types of experimental retainers was investigated with the commercially available everStick Ortho (EST) and stainless-steel (SS) orthodontic retainers. The SS retainers revealed better fatigue resistance; however, nHA/EG retainers showed comparable results to EG and EST retainers among fiber-based retainers. The difference in debonding force was non-significant among glass-fiber-based retainers. The glass-fiber-based retainers exhibited less adhesion of *S. aureus* and *C. albicans* compared to the SS retainers after 48 h and 168 h. The EST group showed significantly lower adhesion compared to EG and nHA/EG. Based on these results, further testing, such as the evaluation of the effect of experimental retainers on periodontal health and in vivo studies, should be conducted.

## Figures and Tables

**Table 1:** Description of control and experimental groups.

Name	Code	Composition	Manufacturer
**Nano-hydroxyapatite (nHA) grafted glass fiber**	nHA/EG	Nano-hydroxyapatite, E-glass fiber, *bis*-GMA, UDMA	Experimental
**Pure E-glass fiber**	EG	E-glass fiber, *bis*-GMA, UDMA	Experimental
**everStick Ortho**	EST	E-glass, PMMA *, *bis*-GMA	StickTech Ltd., Turku, Finland
**3M lingual retainer**	SS	0.032 in. twisted bondable lingual retainer	3M, St. Paul, MN, USA

* PMMA: Polymethylmethacrylate.

**Table 2:** Frequencies (%) of failure types of experimental and control orthodontic retainers after debonding.

Code	Type 1	Type 2	Type 3	Type 4	Combination
**nHA/EG**	0%	10%	45%	0%	45% (Type 3 and 4)
**EG**	0%	30%	40%	0%	30% (Type 3 and 4)
**EST**	0%	20%	50%	0%	30% (Type 3 and 4)
**SS**	50%	50%	0%	0%	0%

Type 1, complete adhesive debonding of the retainer from the tooth surface; Type 2, partial adhesive detachment of the retainer from one of the teeth; Type 3, retainer did not debond from the tooth surface but fractured; Type 4, retainer did not debond from the tooth surface but the overlying composite detached. See Table 1 for a detailed description of the groups.

## Data Availability

Not applicable.

## Supplementary Materials

The following supporting information can be downloaded at: https://www.mdpi.com/article/10.3390/ma15103504/s1. Figure S1: (a) FTIR spectrum of nano-hydroxyapatite grafted glass fibers after silanization with MPS, (b,c) SEM images of silanized E-glass fiber and nano-hydroxyapatite grafted E-glass fiber. Table S1: The summary of data presenting mean and SD values of debonding force and biofilm % of *S. aureus* and *C. albicans* at 48 h and 168 h.
