# Supplementary material for: Preparation of Nano-Apatite Grafted Glass-Fiber-Reinforced Composites for Orthodontic Application: Mechanical and In Vitro Biofilm Analysis
Source: Materials (Basel). 2022 May 13;15(10):3504. doi: 10.3390/ma15103504 (PMC9142944; doi:10.3390/ma15103504)
Supplement: Supplementary file 1 [file materials-15-03504-s001.zip › materials-1697869-supplementary.pdf]

Supplementary File

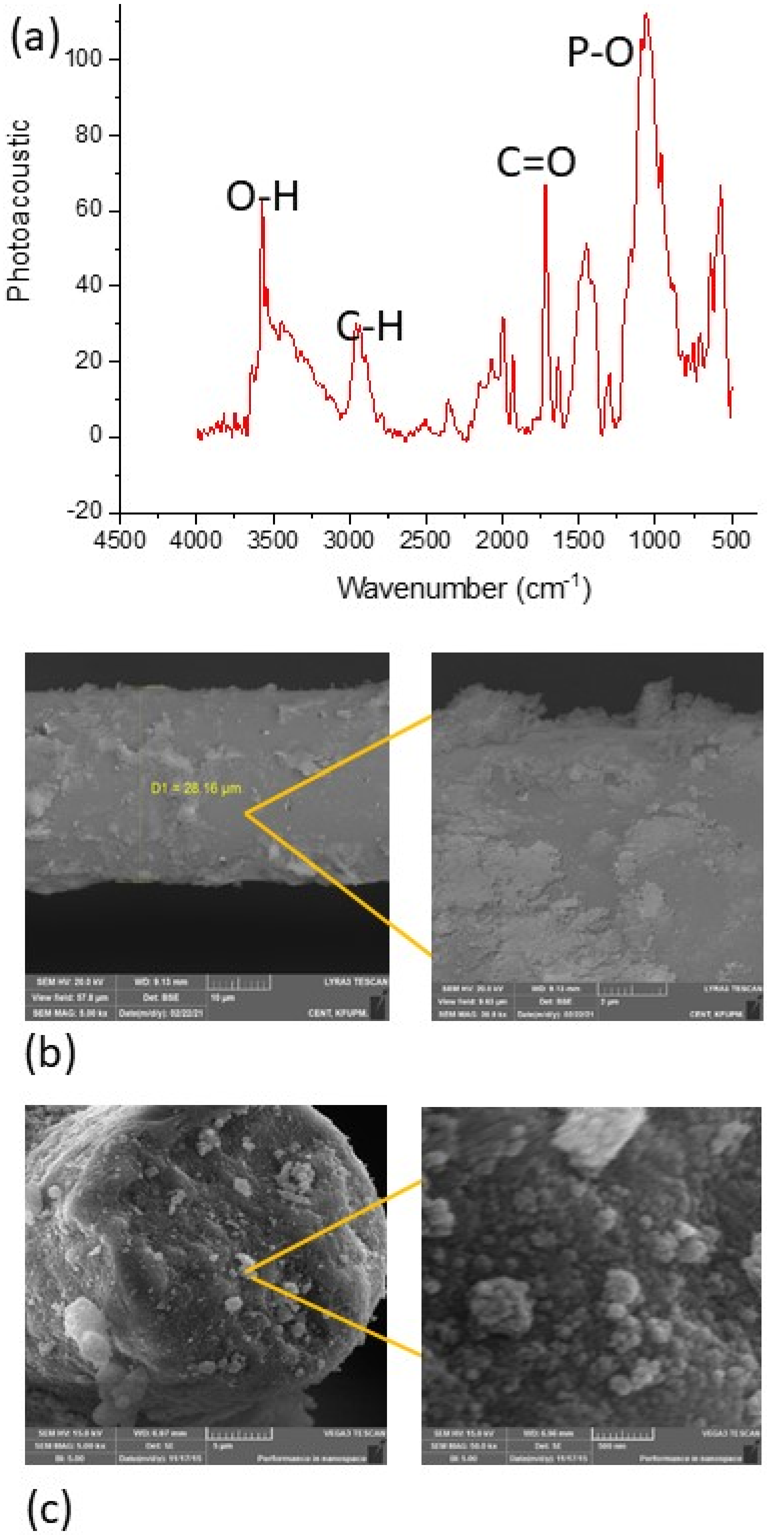

Figure S1 (a) FTIR spectrum of nano-hydroxyapatite grafted glass fibers after silanization with MPS, (b and c) SEM images of silanized E-glass fiber and nano-hydroxyapatite grafted E-glass fiber

Table S1 The summary of data presenting mean and SD values of debonding force and biofilm % of *S. aureus* and *C. albicans* at 48 h and 168 h

**Groups**	**Debonding Force (N)**	***S. aureus* (Biofilm %)**		***C. albicans* (Biofilm %)**	
		**48 hours**	**168 hours**	**48 hours**	**168 hours**
nHA/EG	106.33±28.28	55.33±3.05	73.33±3.05	20.33±0.57	38.06±0.63
EG	103.00±34.34	56.33±3.78	41± 1	18.86±0.55	25.16±2.74
SS	251.81±15.22	100	100	100	100
EST	144.36±28.16	46.66±2.88	33.83±1.52	8.86±0.76	11.33±1.52
